# Pea starch increases the dry matter flow at the distal ileum and reduces the amino acids digestibility in ileal digesta collected after 4 hours postprandial of pigs fed low-protein diets

**DOI:** 10.5713/ab.21.0354

**Published:** 2022-01-03

**Authors:** Junyan Zhou, Lu Wang, Guangxin Yang, Lijie Yang, Xiangfang Zeng, Shiyan Qiao

**Affiliations:** 1 State Key Laboratory of Animal Nutrition, Ministry of Agriculture Feed Industry Centre, China Agricultural University, Beijing 100193, China; 2 Beijing Bio-feed additives Key Laboratory, Beijing 100193, China

**Keywords:** Amino Acid Digestibility, Dry Matter Flow, Low-protein Diets, Pigs, Starch Structure

## Abstract

**Objective:**

The study was aimed to investigate the rules of postprandial changes in intestine digesta dry matter (DM) flow and amino acid digestibility of growing pigs fed low-protein (LP) diets made of different starch.

**Methods:**

Eight barrows (28.8±2.1 kg) with a T-cannula at the distal ileum were randomly allotted to an 8×3 Youden square design. Treatments included: waxy corn starch LP (WLP); corn starch LP (CLP) and pea starch LP (PLP). Diets were given at 08:00 and 20:00. Digesta samples were collected in six 2-h stages from 08:00 to 20:00.

**Results:**

The Cr concentrations of ileal digesta increased and then decreased in WLP and CLP, while increased continuously in PLP as time passed after postprandial (p<0.05). Higher average Cr concentrations (0.78% and 0.84% vs 0.70%; p<0.05) and lower average DM flow (181.1 g/kg and 166.3 g/kg vs 240.3 g/kg; p<0.001) were observed in WLP and CLP, compared with PLP. The apparent ileal digestibility coefficient of most amino acids in WLP and CLP increased compared with that in PLP. No difference in lysine or methionine digestibility was observed. When digesta were collected in 2-h periods, the apparent ileal digestibility coefficient of amino acids did not change over time. When digesta was collected in 4-h periods from 16:00 to 20:00 and 6-h periods from 14:00 to 20:00 (p<0.05), WLP and CLP showed markedly higher amino acid digestibility than PLP

**Conclusion:**

High-amylose slowly digested starch can increase the DM flow at the distal ileum and reduce the apparent ileal digestibility coefficient of amino acids of pigs fed LP diets. Compared with waxy corn starch and corn starch, pea starch reduced the digestibility of amino acids in digesta collected after 4 h postprandial.

## INTRODUCTION

Appropriate modulation of dietary starch profile can enhance nitrogen efficiency via optimizing the synchronization of energy and nitrogen supply in pigs [1,2] and improve digestive dynamics of protein and amino acids in broiler chickens, when offered low-protein (LP) diets [3]. However, these improvements may not be the only positive results of dietary starch profile modulation. Starch and protein are mixed in digesta [4]. Previous studies proved that changes in starch digestion rates caused by dietary starch structure modulation can induce varying degrees of contact between protein and protease, which in turn affects protein digestion and absorption [5,6]. Due to the large amounts of crystalline amino acids supplementation, the nitrogen source in LP diets were markedly changed. However, few studies on the effect of starch structure on nitrogen digestion and absorption efficiency in LP diets were reported.

Furthermore, the digestibility and digestion rate of nutrients are mainly decided by the rate of hydrolysis and digesta transit [7,8]. The hydrolysis rate is closely related to the digesta transit, and both are mainly determined by digesta rheological properties [9]. Research found that dietary starch characteristics directly affect the digesta rheological properties, such as shear stress, storage modulus and gastrointestinal retention time, which determines the dry matter (DM) flow of digesta [10,11]. Elevated digesta DM flow (g/kg DM intake [DMI]) indicates increased digesta nutrients pass through the distal digestive tract, causing a decrease in digestibility [12]. Investigating the time-varying rules of digesta DM flow and dietary nutrients digestibility and exploring the interaction of nutrient digestion are of great significance to the dietary digestion kinetics research.

Kim et al [12,13] explored the postprandial changes in digesta DM flow and amino acids digestibility of growing pigs fed corn, soybean meal and distiller’s dried grains with solubles and recommended a sample collection period for the amino acid digestibility determination. In the current study, we used different kinds of purified starch to regulate the dietary starch structure, and the sources of protein and fiber in each treatment group were the same, which avoided the difference in other nutrients caused using different feed ingredients. Previous studies have shown that waxy corn starch containing high amylopectin has a fast digestion rate, while pea starch containing high amylose has a slow one in the proximal intestine [14]. We speculate that slow digestion of starch will hinder the digestion and absorption of dietary nutrients, resulting in more DM flowing out of the terminal ileum and reduced amino acid digestibility. The objectives of the present study were to explore the postprandial time-varying changes rules of DM flow and amino acids digestibility in distal ileal digesta of growing pigs fed LP diets and to investigate the influence of starch structure on both two matters.

## MATERIALS AND METHODS

### Animal care

The present experiment was approved by the China Agricultural University Animal Care and Use Committee (Beijing, AW12401202-1-1). All pigs and experimental supplies were provided by the Fengning Swine Research Unit of China Agricultural University (Academician Workstation in Chengdejiuyun Agricultural and Livestock Co., Ltd).

### Animals and diets

Eight crossbred (Duroc×Landrace×Yorkshire) barrows (28.8 ±2.1 kg) fitted with a T-cannula at the terminal ileum were randomly allotted to 1 of 3 experimental diets in a 3-period Youden square design which resulted in 8 observations per dietary treatment [15]. Dietary treatments included: a waxy corn starch LP diet (WLP); a corn starch LP diet (CLP) and a pea starch LP diet (PLP). Experimental diets were formulated according to the net energy system, and other dietary nutrients were formulated. Pigs were housed individually in stainless-steel metabolism crates (1.4 m×0.7 m×0.6 m) based on the National Research Council [16], with slight modifications (Table 1).

### Feeding and sample collection

Daily feed allotment (about 4% of body weight) was divided into two equal meals given to each pig at 08:00 and 20:00 during the experimental period. All the pigs had access to fresh water *ad libitum*. The temperature was maintained at 23°C±2°C. Humidity varied from 55% to 65% during the experiment.

Each experiment period consisted of 7 days (4 days for adaptation to diets and 3 days for sample collection). During sample collection period, ileal digesta samples were collected in six 2-h stages from 08:00 to 20:00 (in detail, ileal digesta samples were collected from postprandial 0 to 2 h; 2 to 4 h; 4 to 6 h; 6 to 8 h; 8 to 10 h; and 10 to 12 h). A 300-mL plastic bag with 5 g chlortetracycline in it was attached to the cannula barrel using an elastic plastic rope. Bags were removed at least once every 30 min, and immediately stored at −20°C. Low-temperature storage and antibiotics supplementation were used to weaken microbial degradation of nutrients in digesta. After collection period, samples from each pig were mixed across the 3 collection days within collection stages. In summary, 6 digesta samples were collected from each pig in each 3-day collection period, and each sample representing a 2-h collection stage.

### Chemical analyses

Frozen digesta samples were placed at room temperature to thaw and then weighed. After adequate mixture, a subsample from each 2-h collection stage digesta sample was weighed, lyophilized, weighed, and finely ground. The analysis of feed and digesta samples was conducted in duplicate. The analysis of DM, crude protein, total calcium, and total phosphorus concentrations in feed and digesta samples was conducted according to the Association of Official Analytical Chemists procedures [17]. Samples of diets and ileal digesta were hydrolyzed with 6-*N* HCl for 24 h at 110°C before analysis for 15 amino acids with an Amino Acid Analyzer (Hitachi L-8900, Tokyo, Japan). The concentrations of methionine and cysteine were measured according to a common method. Briefly, samples were subjected to performic acid oxidation and hydrolyzed with 7.5-*N* HCl for 24 h at 110°C and determined using an Amino Acid Analyzer (Hitachi L-8800, Japan). Tryptophan concentrations in feed and digesta samples were determined using High Performance Liquid Chromatography (Agilent 1200 Series, Santa Clara, CA, USA) after NaOH hydrolysis for 22 h at 110°C. Chromium concentrations measurement was conducted using a Polarized Zeeman Atomic Absorption Spectrometer (Hitachi Z2000, Japan). Starch content in diet and ileal digesta was determined with commercial assay kits (Nanjing Jiancheng Bioengineering Institute, Nanjing, China).

### Calculation and statistical analysis

The ileal Cr flow and the ileal DM flow present the output of Cr and DM in ileal digesta, respectively. These result values were calculated for 4-, 6-, and 12-h collection stages on the basis of the following equation:


Concentrations (%)=Σ[concentration (%)×sample (g)]/Σsample (g)

Where sample (g) means the weight of the ileal digesta sample after freeze-drying.

The ileal DM flows were calculated according to the following equation:


$$\text{Ileal DM flow }(\text{g}/\text{kg DMI})={\text{Cr}}_{\text{diet}}/{\text{Cr}}_{\text{ileal}}$$

Where Cr_diet_ means dietary Cr concentration (g/kg DM), and Cr_ileal_ means Cr concentration in ileal digesta (g/kg DM).

Values for apparent ileal digestibility coefficient of amino acids were calculated as previously described [18].

Data were obtained with each 2-, 4-, 6-, and 12-h collection stages as one experimental unit. Experimental repeated measure data was analyzed using MIXED procedures of SAS version 9.4 (SAS Institute, Cary, NC, USA). The differences were considered significant when p<0.05, and the differences were considered highly significant when p<0.001. The analysis results of analysis of variance method were used to describe the differences between treatment groups.

## RESULTS AND DISCUSSION

Much research has reported the impact of starch structure modulation on pig husbandry, such as changing dietary glycemic properties, regulating growth performance and carcass traits [2,19]. However, few studies analyzed the digestion kinetics and nutrient utilization of LP diets consisting of different starch structures. We innovatively explored the effect of starch structures on DM flow and amino acids digestibility in distal ileal digesta under LP condition. Our data indicated that high-amylose slowly digested starch can increase the DM flow at the distal ileum and reduce the apparent ileal digestibility coefficient of amino acids.

The waxy corn starch, corn starch and pea starch used in this experiment are generally regarded as high-digestion rate, medium-digestion rate, and low-digestion rate starch, respectively [14]. The starch digestion rate is mainly determined by the crystal structure, amylose/amylopectin ratio, particle size, porosity, and viscosity [20]. The diameter of waxy corn starch particles is much smaller than that of corn starch and pea starch, and the particles are mostly cubic in shape, which enlarges the contact area with enzymes [21]. Enzymes hydrolyze large granules (pea starch) mainly by producing considerable surface corrosion with deep cylindrical channels on the radial axis of the granules. For small granules (waxy corn starch), solubilization and corrosion occur simultaneously on the granules surface. After hydrolysis, a more fragmented appearance appears in smaller granules suggesting they are more fragile than larger ones [22]. The previous analysis results of our laboratory showed that the amylopectin contents of waxy corn starch, corn starch and pea starch were about 100%, 70%, and 40%, respectively. The branched structure of amylopectin could induce greater gelatinization and a greater surface area for digestive enzymes and is subsequently more rapidly absorbed than amylose [21]. Reticulated amylopectin is organized in the helix conformation with linear amylose to set up crystalline structures. According to the difference of crystalline structure, starch is divided into three types: A, B, and C. In A-type crystalline starch, glucose is packed tightly. While in B-type crystalline starch, glucose is loosely packed, which gives space to water molecules between the branches. C-type crystalline starch is composed of A and B-type starch. Pea starch, as a C-type crystalline starch, is more difficult to digest than waxy corn starch and corn starch (A-type crystalline starch) due to the water molecules contained in it [23]. Moreover, in most instances, a high dietary proportion of amylose will increase the viscosity of the digesta [24]. The high viscosity of digesta in PLP treatment can hinder the mixture of nutrients with digestive enzymes, and impair digestion [24,25].

To achieve the objective of the present study, a T-cannula was installed about 10 cm proximal to the ileo-blind junction by surgery for ileal digesta collection. In the experiment, when pigs were fed LP diets based on waxy corn starch (p<0.001) and corn starch (p<0.001), the Cr concentrations of ileal digesta collected in 2-h periods from 08:00 to 20:00 increased and then decreased (Table 2). This observation suggests that the changes in the ingredients of ileal digesta collected from different postprandial periods do exist, which has also been reported in previous research [26]. The maximum concentration of Cr in ileal digesta was observed at postprandial 6 to 10 h in the present study and this result is later than those observed by Kim et al [12], Livingstone et al [27] and Graham and Åman [26]. Kim et al [12] found that the Cr concentration of ileal digesta collected in 2-h periods increased linearly from 08:00 to 20:00 when pigs were fed the purified N-free diet. In this study, although each treatment group also used purified starch as the starch source, soybean meal was used to provide nitrogen for the treatment diets, which may lead to the difference between the two studies.

When pigs were fed WLP diet, the concentration of Cr in ileal digesta samples collected in postprandial 4 to 6 h, 6 to 8 h, and 8 to 10 h was higher than the concentration of Cr in ileal digesta samples collected in postprandial 0 to 2 h (p<0.05). Pigs fed CLP diet showed increased concentration of Cr in ileal digesta samples collected in postprandial 6 to 8 h and 8 to 10 h, compared with those collected in postprandial 0 to 2 h and 2 to 4 h (p<0.05). Chromium content in ileal digesta of the PLP fed pigs increased until 6 to 8 h after a meal (p< 0.05). There was no difference in Cr concentration in ileal digesta samples of the PLP fed pigs collected in postprandial 4 to 6 h, 6 to 8 h, 8 to 10 h, and 10 to 12 h. It is worth noting that whether pigs fed WLP, CLP, or PLP diets, the concentration of Cr in ileal digesta samples collected in 4-h period from postprandial 0 to 4 h was less than those collected in 4-h period from postprandial 4 to 8 h and from postprandial 8 to 12 h (p<0.05). When the collection time of digesta is divided into the first 6 h and the second, the concentration of Cr in the first 6 h is lower than that in the latter (p<0.001). This situation happened in all three treatment groups.

In most sample collection periods, the concentration of Cr in pigs fed WLP and CLP diets was higher than that in pigs fed PLP diet in the corresponding collection period (p<0.05). These changes were mainly due to differences in DM flow because the DM flow had an opposite pattern compared with the concentration of Cr in the ileal digesta samples. During the whole 12 h of sample collection, the average concentration of Cr in ileal digesta and the average DM flow at the distal ileum were 0.78% and 181.1 g/kg DMI from pigs fed the WLP diet, 0.84% and 166.3 g/kg DMI from pigs fed CLP diet and 0.70% and 240.3 g/kg DMI from pigs fed PLP diet. Statistics proved that compared with WLP and CLP groups, PLP treatment group showed a decreased average Cr concentration of ileal digesta (p<0.05) and increased average DM flow at the distal ileum (p<0.001). This result suggested that the chemical components of ileal digesta depend on the pattern of dietary starch structure. Improved dietary amylose proportion induces increased DM flows through the foregut and decreased digesta Cr concentration. Previous research proved that the high dietary fiber content can increase the content of DM flows pass the small intestine but can decrease the concentration of Cr in the digesta and nutrient digestibility [12,27]. Therefore, the results of the present experiment possibly demonstrate that amylose, like fiber, itself is not easy to digest and hinders the digestion and absorption of other nutrients in digesta [28]. On the other hand, as shown in Table 3, the ileal digestibility coefficient of starch in PLP group was 6% lower than that in WLP and CLP groups, which may suggest that the content of resistant starch in PLP group was higher. A study has confirmed that resistant starch stimulates endogenous loss [29]. Therefore, in this study, the reduced apparent ileal digestibility coefficient of amino acid in the PLP group may also be due to the increase of endogenous amino acid loss.

The data for the apparent ileal digestibility coefficient of amino acids further confirmed this hypothesis. The apparent ileal digestibility coefficient of threonine, tryptophan, leucine, valine, histidine, arginine, serine, glycine, aspartate, and total amino acids in pigs fed WLP and CLP diets was higher than that in pigs fed PLP diet (p<0.001; Table 3). The apparent ileal digestibility coefficient of glutamate, proline, alanine, tyrosine and cystine in pigs fed CLP diets was markedly increased compared with that in pigs fed PLP diet (p< 0.05). Compared with WLP treatment group, the PLP treatment groups showed dramatically decreased apparent ileal digestibility coefficient of isoleucine and phenylalanine (p< 0.05). Previous studies (unpublished data) have proved that the dietary starch profile modulation (use equal amounts of waxy corn starch and corn starch as the starch source) improved the synchronization of the energy and nitrogen supply of LP diets, thereby optimizing the nitrogen efficiency in growing pigs. The results of the current experiment suggested that alteration in dietary starch structure can also change the digestibility of amino acids. The outcomes of these two studies are not contradictory, because there is no significant difference in amino acids digestibility between LP diets consisted of waxy corn starch and corn starch.

What draws our attention was that the apparent ileal digestibility coefficient of lysine or methionine was not different among treatments (Table 3). These results may be because the experimental diets in the current study were all LP diets, and large amounts of crystalline amino acids were supplemented to satisfy the amino acid nutritional requirement of pigs [30]. Especially since crystalline lysine and methionine, their content in the diets was 0.53% and 0.25%, respectively, account for almost half of the dietary digestible lysine and methionine content. Different from intact protein, crystalline amino acids exist in monomer form and can be directly absorbed by the intestines without digestion, and their digestibility is almost 100% [31]. Therefore, even though the digestibility of lysine and methionine derived from the feed materials among treatments may be quite different, there will be no significant difference in the apparent ileal digestibility coefficient of these two amino acids.

The apparent ileal digestibility coefficient of amino acid is described as the net disappearance of dietary amino acids from the digestive tract proximal to the distal ileum [18]. The term “apparent” gives an emphasis to undigested dietary amino acids and endogenous amino acids that were secreted into the digestive tract and not reabsorbed ahead of the distal ileum, which leads to the total ileal outflow of amino acids. Because apparent ileal digestibility coefficient is influenced by endogenous amino acid loss, it leads to a great defect with the use of apparent ileal digestibility coefficient in diet formulation due to the insufficiency of additivity of apparent ileal digestibility coefficient in mixtures of feed ingredients [32]. However, the aim of the present study was to explore the postprandial time-varying changes rules of DM flow and amino acids digestibility in distal ileal digesta of growing pigs fed LP diets and investigate the influence of starch structure on both two matters. It did not involve the accurate evaluation of the feed ingredients’ nutritional value and the formulas. Moreover, the endogenous losses change greatly among pigs, and differences in endogenous loss may be observed between different laboratories even though the experimental conditions are closely controlled [33]. Therefore, in the present study, the endogenous amino acid loss has not been determined. Incidentally, although the loss of endogenous amino acids comes from animal intestines, not undigested and absorbed dietary amino acids, the increase of endogenous amino acid loss also means that more amino acids are wasted.

We show the apparent ileal digestibility coefficient of four key essential and non-essential amino acids based on the collection of ileal digesta during different time periods in Table 4 and 5, respectively. Because the change rules in the digestibility of different amino acids have strong similarities, we have not shown all the amino acids, which is considered unnecessary and complicated. When digesta was collected in 2-h periods from postprandial 0 to 12 h, the apparent ileal digestibility coefficient of essential and non-essential amino acids in each treatment group did not change significantly over time. Pigs fed CLP diet showed increased apparent digestibility of threonine, leucine, glycine, aspartate, and glutamate in ileal digesta samples collected in postprandial 4 to 8 h and 8 to 12 h, compared with that collected in postprandial 0 to 4 h (p<0.05). Besides, apparent digestibility of threonine, leucine, glycine, and alanine in ileal digesta samples collected in postprandial 6 to 12 h increased markedly compared with that collected in postprandial 0 to 6 h of CLP treatment (p< 0.05). When pigs fed WLP diets, the apparent digestibility in ileal digesta samples collected in 4-h period from postprandial 0 to 4 h was less than those collected in 4-h period from postprandial 8 to 12 h (p<0.05). Moreover, in WLP treatment group, when the collection time of digesta is divided into the first 6 h and the second, the apparent ileal digestibility coefficient of glycine and alanine in the first 6 h is lower than that in the second (p<0.05).

Although the apparent ileal digestibility coefficient of amino acids in each treatment did not change significantly with time when digesta was collected in 2-h periods, which may be because of differences within the group. We still find that the digestibility of amino acids tended to increase over time or decrease after 10 h postprandial. These results are different from those of Kim et al [13] where the maximum digestibility values were observed 4 to 8 h after the meal. The peak of amino acid digestibility shown in this study was at least 4 h later than that in Kim et al [13]. This may be due to the difference in the diet types used in the two studies. Compared with the diets made of corn, soybean meal and corn dry distillers grains with solubles respectively, the semi pure diets in the present experiment mainly used purified starch as starch source and alfalfa meal and cellulose acetate as fiber source.

When comparing differences in amino acids digestibility of ileal digesta during different time periods between groups, the situation is similar to the comparison in Cr concentration. In general, essential (except lysine and methionine) and non-essential amino acids digestibility in WLP and CLP treatments was higher than that in PLP treatment during most time periods. When digesta was collected in 2-h periods and 4-h periods from postprandial 0 to 4 h, the apparent ileal digestibility coefficient of essential (except threonine) and non-essential amino acids between treatment groups did not differ. There was no difference in lysine and methionine digestibility between treatments, the only exception is that lysine digestibility in WLP and CLP treatments was higher than that in PLP treatment in ileal digesta samples collected during postprandial 8 to 10 h (p<0.001).

## CONCLUSION

When the digesta samples were collected in six 2-h periods, waxy corn starch (high amylopectin) and corn starch (medium amylopectin) induced the digesta DM flow to decrease and then increase, and pea starch (high amylose) caused the digesta DM flow to decrease and then stay on a plateau. Throughout digesta collection period, compared with waxy corn starch and corn starch, pea starch increased digesta DM flow and reduced the apparent ileal digestibility of dietary amino acids. Differences in amino acids digestibility between groups were mainly reflected in the digesta collected after 4 h postprandial.

## Figures and Tables

**Table 1:** Ingredients and nutrient composition of experimental diets (as-fed basis)

Item	WLP^1)^	CLP^1)^	PLP^1)^
Ingredient (%)			
Waxy corn starch	57.51	-	-
Corn starch	-	57.51	-
Pea starch	-	-	57.51
Soybean meal, 44% crude protein	20.68	20.68	20.68
Wheat bran	8.00	8.00	8.00
Alfalfa meal	6.00	6.00	6.00
Cellulose acetate	2.00	2.00	2.00
Dicalcium phosphate	1.93	1.93	1.93
Limestone	0.22	0.22	0.22
Salt	0.30	0.30	0.30
Premix^2)^	1.00	1.00	1.00
Chromic oxide	0.40	0.40	0.40
L-Lys HCL, 78.8%	0.53	0.53	0.53
DL-Met	0.25	0.25	0.25
L-Trp	0.04	0.04	0.04
L-Thr	0.23	0.23	0.23
L-Val	0.24	0.24	0.24
L-Leu	0.29	0.29	0.29
L-Ile	0.10	0.10	0.10
L-Phe	0.16	0.16	0.16
L-His	0.12	0.12	0.12
Calculated composition			
Net energy (kcal/kg)	2550	2550	2550
SID Lys (%)	1.01	1.01	1.01
SID SAA (%)	0.58	0.58	0.58
SID Trp (%)	0.18	0.18	0.18
SID Thr (%)	0.63	0.63	0.63
SID Val (%)	0.63	0.63	0.63
SID Leu (%)	1.03	1.03	1.03
SID Ile (%)	0.57	0.57	0.57
SID Phe (%)	0.59	0.59	0.59
SID His (%)	0.34	0.34	0.34
SID Arg (%)	0.69	0.69	0.69
Analyzed composition (%)			
Dry matter	89.78	90.01	88.98
Crude protein	13.00	13.31	13.25
Crude fiber	3.75	3.77	3.75
Total calcium	0.67	0.67	0.66
Total phosphorus	0.56	0.56	0.57
Lys	1.13	1.18	1.15
Met	0.42	0.41	0.39
Thr	0.69	0.65	0.69
Trp	0.20	0.20	0.19
Leu	1.26	1.30	1.32
Ile	0.58	0.56	0.60
Val	0.74	0.69	0.69
Phe	0.66	0.62	0.65
His	0.39	0.41	0.40
Arg	0.75	0.78	0.75
Cys	0.20	0.21	0.18
Asp	1.38	1.45	1.38
Ser	0.62	0.58	0.64
Glu	1.99	1.87	1.98
Pro	0.56	0.55	0.61
Gly	0.60	0.67	0.62
Ala	0.62	0.56	0.61
Tyr	0.40	0.38	0.45

SID, standardized ileal digestible.

1) WLP, waxy corn starch low-protein diet; CLP, corn starch low-protein diet; PLP, pea starch low-protein diet.

2) Premix provided the following per kg of complete diet for growing pigs: vitamin A, 5,512 IU; vitamin D_3_, 2,200 IU; vitamin E, 64 IU; vitamin K_3_, 2.2 mg; vitamin B_12_, 27.6 μg; riboflavin, 5.5 mg; pantothenic acid, 13.8 mg; niacin, 30.3 mg; choline chloride, 551 mg; Mn, 40 mg (MnSO_4_); Fe, 100 mg (FeSO_4_·H_2_O); Zn, 100 mg (ZnSO_4_); Cu, 100 mg (CuSO_4_·5H_2_O); I, 0.3 mg (KI); Se, 0.3 mg (Na_2_SeO_3_).

**Table 2:** Concentrations of Cr and the flow of DM (g/kg DMI) in ileal digesta samples during different postprandial time periods from pigs fed low-protein diets consisted of different starch

Time after feeding	Cr (%)			SEM	p-value	DM flow (g/kg DMI)			SEM	p-value
	
	WLP^1)^	CLP^1)^	PLP^1)^			WLP^1)^	CLP^1)^	PLP^1)^		
0 to 2 h	0.56^b,Z^	0.69^b,Y^	0.61^b,ZY^	0.03	0.042	173.1^Z^	170.5^Z^	263.8^a,Y^	16.8	<0.001
2 to 4 h	0.76^ab,Y^	0.73^b,Y^	0.56^b,Z^	0.04	<0.001	183.1^Z^	184.5^Z^	292.3^a,Y^	22.3	<0.001
4 to 6 h	0.85^a,Y^	0.85^ab,Y^	0.73^a,Z^	0.03	0.031	160.9^Z^	157.0^Z^	222.3^ab,Y^	11.4	0.025
6 to 8 h	0.91^a,Y^	0.98^a,Y^	0.74^a,Z^	0.03	0.006	152.3^Z^	139.4^Z^	214.7^b,Y^	18.5	0.037
8 to 10 h	0.91^a,ZY^	1.02^a,Y^	0.74^a,Z^	0.04	<0.001	152.2^Z^	136.5^Z^	214.8^b,Y^	12.9	0.009
10 to 12 h	0.76^ab^	0.83^ab^	0.73^a^	0.03	0.047	178.7	169.4	229.5^ab^	23.7	0.147
SEM	0.05	0.05	0.04			29.8	22.4	18.4		
p-value	0.008	0.004	0.033			0.872	0.514	0.026		
Linear	0.015	0.012	0.027			0.452	0.625	0.042		
Quadratic	<0.001	<0.001	0.008			0.248	0.382	<0.001		
0 to 4 h	0.62^b^	0.70^b^	0.60^b^	0.05	0.421	225.9^a,Z^	196.0^a,Z^	266.8^a,Y^	11.5	0.006
4 to 8 h	0.87^a,Y^	0.96^a,Y^	0.73^a,Z^	0.04	0.021	138.3^b,Z^	134.0^b,Z^	220.7^b,Y^	13.4	<0.001
8 to 12 h	0.81^a,YZ^	0.89^a,Y^	0.73^a,Z^	0.04	0.033	170.0^b,Z^	158.9^b,Z^	223.1^b,Y^	12.1	<0.001
SEM	0.03	0.06	0.03			11.3	11.7	12.9		
p-value	0.008	0.031	0.034			0.010	0.022	0.029		
0 to 6 h	0.75^b^	0.74^b^	0.66^b^	0.04	0.061	189.4^a,Z^	184.9^a,Z^	247.1^Y^	18.1	0.022
6 to 12 h	0.85^a,ZY^	0.93^a,Y^	0.73^a,Z^	0.04	0.007	163.1^b,Z^	150.3^b,Z^	223.8^Y^	14.9	0.006
SEM	0.02	0.03	0.02			5.2	9.6	6.1		
p-value	<0.001	<0.001	<0.001			<0.001	<0.001	0.031		
0 to 12 h	0.78^Y^	0.84^Y^	0.70^Z^	0.02	0.042	181.1^Z^	166.3^Z^	240.3^Y^	14.2	<0.001

Each least square mean represents 8 observations for each treatment. Data from collection for 4, 6, or 12 h were calculated based on collections in the six 2-h periods.

DM, dry matter; DMI, dry matter intake; SEM, standard error of the mean.

1) WLP, waxy corn starch low-protein diet; CLP, corn starch low-protein diet; PLP, pea starch low-protein diet.

a,b Means in the same column with different superscripts differ among collection times (p≤0.05).

Y,Z Means in the same row with different superscripts differ among treatments (p≤0.05).

**Table 3:** Apparent ileal digestibility coefficient of amino acids and starch in low-protein diets consisted of different starch fed to growing pigs

Items	WLP^1)^	CLP^1)^	PLP^1)^	SEM	p-value
Essential amino acids					
Lysine	0.74	0.77	0.73	0.02	0.214
Methionine	0.94	0.94	0.93	0.02	0.676
Threonine	0.75^a^	0.78^a^	0.69^b^	0.02	<0.001
Tryptophan	0.76^a^	0.76^a^	0.69^b^	0.01	<0.001
Isoleucine	0.77^a^	0.72^ab^	0.60^b^	0.02	<0.001
Leucine	0.78^a^	0.79^a^	0.70^b^	0.01	<0.001
Valine	0.80^a^	0.80^a^	0.72^b^	0.02	<0.001
Phenylalanine	0.83^a^	0.80^ab^	0.77^b^	0.01	<0.001
Histidine	0.83^a^	0.80^a^	0.72^b^	0.02	<0.001
Arginine	0.78^a^	0.81^a^	0.71^b^	0.02	<0.001
Non-essential amino acids					
Serine	0.78^a^	0.78^a^	0.68^b^	0.01	<0.001
Glutamate	0.77^ab^	0.82^a^	0.72^b^	0.02	<0.001
Proline	0.77^ab^	0.80^a^	0.70^b^	0.03	0.008
Glycine	0.78^a^	0.79^a^	0.70^b^	0.02	<0.001
Alanine	0.77^ab^	0.80^a^	0.74^b^	0.01	0.011
Tyrosine	0.77^ab^	0.80^a^	0.73^b^	0.02	0.012
Aspartate	0.77^a^	0.77^a^	0.68^b^	0.01	<0.001
Cystine	0.75^ab^	0.77^a^	0.70^b^	0.02	0.023
Total amino acids	0.78^a^	0.80^a^	0.69^b^	0.01	<0.001
Starch	0.98	0.98	0.92	0.04	0.363

Each least square mean represents 8 observations for each treatment.

SEM, standard error of the mean.

1) WLP, waxy corn starch low-protein diet; CLP, corn starch low-protein diet; PLP, pea starch low-protein diet.

a,b Means in the same row with different superscripts differ (p≤0.05).

**Table 4:** Apparent ileal digestibility coefficient of key essential amino acids calculated based on collection of ileal digesta during different postprandial time periods from pigs fed low-protein diets consisted of different starch

Time after feeding	Lysine					Methionine					Threonine					Leucine				
			
	WLP^1)^	CLP^1)^	PLP^1)^	SEM	p-value	WLP^1)^	CLP^1)^	PLP^1)^	SEM	p-value	WLP^1)^	CLP^1)^	PLP^1)^	SEM	p-value	WLP^1)^	CLP^1)^	PLP^1)^	SEM	p-value
0 to 2 h	0.72	0.78	0.73	0.03	0.221	0.93	0.94	0.94	0.01	0.901	0.72^YZ^	0.77^Z^	0.65^Y^	0.03	0.021	0.74	0.77	0.71	0.03	0.323
2 to 4 h	0.75	0.65	0.73	0.04	0.453	0.95	0.93	0.94	0.01	0.332	0.75	0.71	0.69	0.04	0.624	0.77	0.71	0.70	0.04	0.516
4 to 6 h	0.72	0.81	0.76	0.03	0.132	0.91	0.94	0.93	0.03	0.718	0.74	0.80	0.72	0license-.03	0.146	0.76^Z^	0.81^Z^	0.69^Y^	0.02	0.041
6 to 8 h	0.78	0.79	0.75	0.03	0.348	0.94	0.95	0.93	0.01	0.267	0.77^Z^	0.79^Z^	0.70^Y^	0.02	0.041	0.81^Z^	0.79^Z^	0.71^Y^	0.02	0.038
8 to 10 h	0.80^Z^	0.82^Z^	0.63^Y^	0.02	<0.001	0.94	0.94	0.93	0.01	0.801	0.78^Z^	0.81^Z^	0.63^Y^	0.02	<0.001	0.81^Z^	0.83^Z^	0.63^Y^	0.02	<0.001
10 to 12 h	0.71	0.76	0.76	0.05	0.735	0.94	0.94	0.94	0.01	0.953	0.74	0.81	0.73	0.03	0.233	0.78^Z^	0.82^Z^	0.71^Y^	0.02	0.024
SEM	0.04	0.06	0.05			0.03	0.01	0.02			0.04	0.04	0.05			0.03	0.04	0.05		
p-value	0.552	0.393	0.342			0.792	0.708	0.912			0.763	0.201	0.523			0.541	0.224	0.877		
Linear	0.347	0.491	0.548			0.388	0.512	0.821			0.549	0.104	0.290			0.832	0.125	0.767		
Quadratic	0.212	0.718	0.539			0.444	0.782	0.387			0.722	0.209	0.655			0.510	0.283	0.375		
0 to 4 h	0.74	0.74	0.73	0.03	0.684	0.94	0.94	0.94	0.01	0.821	0.73	0.74^b^	0.67	0.03	0.100	0.76	0.74^b^	0.71	0.03	0.394
4 to 8 h	0.75	0.80	0.75	0.03	0.138	0.92	0.94	0.93	0.02	0.555	0.75^YZ^	0.80^a,Z^	0.71^Y^	0.03	0.020	0.79^Z^	0.81^a,Z^	0.70^Y^	0.02	<0.001
8 to 12 h	0.74	0.79	0.70	0.04	0.374	0.94	0.94	0.93	0.01	0.714	0.76^YZ^	0.81^a,Z^	0.69^Y^	0.03	0.013	0.79^Z^	0.82^a,Z^	0.68^Y^	0.03	0.023
SEM	0.01	0.02	0.03			0.01	0.01	0.02			0.02	0.02	0.02			0.02	0.02	0.02		
p-value	0.653	0.116	0.315			0.510	0.580	0.768			0.509	0.033	0.534			0.242	0.008	0.326		
0 to 6 h	0.73	0.76	0.74	0.02	0.522	0.93	0.94	0.93	0.01	0.742	0.74^YZ^	0.76^b,Z^	0.68^Y^	0.02	0.033	0.77	0.76^b^	0.71	0.02	0.073
6 to 12 h	0.76	0.79	0.72	0.03	0.265	0.94	0.95	0.93	0.01	0.610	0.76^Z^	0.81^a,Z^	0.70^Y^	0.02	0.014	0.80^Z^	0.81^a,Z^	0.69^Y^	0.02	0.022
SEM	0.02	0.03	0.02			0.01	0.01	0.02			0.02	0.02	0.02			0.01	0.01	0.02		
p-value	0.512	0.718	0.517			0.442	0.818	0.766			0.572	0.054	0.327			0.219	0.032	0.714		
0 to 12 h	0.74	0.77	0.73	0.02	0.213	0.94	0.94	0.93	0.02	0.666	0.75^Z^	0.78^Z^	0.69^Y^	0.02	<0.001	0.78^Z^	0.79^Z^	0.70^Y^	0.01	<0.001

Each least square mean represents 8 observations for each treatment. Data from collection for 4, 6, or 12 h were calculated based on collections in the six 2-h periods.

SEM, standard error of the mean.

1) WLP, waxy corn starch low-protein diet; CLP, corn starch low-protein diet; PLP, pea starch low-protein diet.

a,b Means in the same column with different superscripts differ among collection times (p≤0.05).

Y,Z Means in the same row with different superscripts differ among treatments (p≤0.05).

**Table 5:** Apparent ileal digestibility coefficient of key non-essential amino acids calculated based on collection of ileal digesta during different postprandial time periods from pigs fed low-protein diets consisted of different starch

Time after feeding	Glycine					Alanine					Aspartate					Glutamate				
			
	WLP^1)^	CLP^1)^	PLP^1)^	SEM	p-value	WLP^1)^	CLP^1)^	PLP^1)^	SEM	p-value	WLP^1)^	CLP^1)^	PLP^1)^	SEM	p-value	WLP^1)^	CLP^1)^	PLP^1)^	SEM	p-value
0 to 2 h	0.72	0.76	0.68	0.03	0.324	0.74	0.78	0.76	0.02	0.324	0.74	0.76	0.70	0.03	0.298	0.72	0.82	0.73	0.04	0.064
2 to 4 h	0.77	0.74	0.68	0.03	0.084	0.74	0.77	0.73	0.04	0.679	0.76	0.70	0.69	0.03	0.373	0.75	0.74	0.70	0.04	0.713
4 to 6 h	0.77	0.79	0.72	0.03	0.142	0.77	0.81	0.76	0.02	0.260	0.76^Z^	0.80^Z^	0.66^Y^	0.03	0.052	0.77	0.86	0.76	0.04	0.056
6 to 8 h	0.81^Z^	0.83^Z^	0.72^Y^	0.02	0.018	0.80	0.80	0.74	0.03	0.342	0.80^Z^	0.78^YZ^	0.68^Y^	0.04	0.042	0.81^Z^	0.84^Z^	0.72^Y^	0.02	0.021
8 to 10 h	0.83^Z^	0.83^Z^	0.68^Y^	0.03	<0.001	0.82^Z^	0.82^Z^	0.68^Y^	0.02	<0.001	0.82^Z^	0.80^Z^	0.60^Y^	0.02	<0.001	0.83^Z^	0.86^Z^	0.65^Y^	0.02	<0.001
10 to 12 h	0.81^Z^	0.82^Z^	0.74^Y^	0.02	<0.001	0.79	0.83	0.76	0.03	0.154	0.76^YZ^	0.81^Z^	0.67^Y^	0.02	0.015	0.75	0.84	0.76	0.05	0.564
SEM	0.04	0.04	0.03			0.03	0.02	0.05			0.04	0.04	0.05			0.04	0.05	0.05		
p-value	0.121	0.217	0.593			0.472	0.456	0.649			0.542	0.300	0.682			0.363	0.088	0.504		
Linear	0.452	0.188	0.403			0.871	0.348	0.224			0.832	0.266	0.509			0.841	0.551	0.259		
Quadratic	0.232	0.444	0.221			0.409	0.505	0.574			0.472	0.328	0.293			0.382	0.377	0.329		
0 to 4 h	0.75^b^	0.75^b^	0.68	0.02	0.009	0.74	0.78	0.74	0.02	0.362	0.75	0.74^b^	0.70	0.03	0.222	0.72	0.78^b^	0.74	0.03	0.077
4 to 8 h	0.79^ab,Z^	0.81^a,Z^	0.72^Y^	0.02	<0.001	0.78	0.81	0.75	0.02	0.104	0.78^Z^	0.79^a,Z^	0.68^Y^	0.03	<0.001	0.78^YZ^	0.85^a,Z^	0.73^Y^	0.02	<0.001
8 to 12 h	0.82^a,Z^	0.82^a,Z^	0.72^Y^	0.02	<0.001	0.80^Z^	0.83^Z^	0.73^Y^	0.02	<0.001	0.78^Z^	0.81^a,Z^	0.65^Y^	0.02	<0.001	0.78^YZ^	0.85^a,Z^	0.71^Y^	0.03	<0.001
SEM	0.02	0.01	0.02			0.03	0.03	0.02			0.02	0.01	0.02			0.03	0.02	0.02		
p-value	0.043	0.032	0.328			0.510	0.280	0.808			0.572	0.018	0.209			0.282	0.038	0.566		
0 to 6 h	0.75^b,Z^	0.77^b,Z^	0.69^Y^	0.02	<0.001	0.75^b^	0.79^b^	0.75	0.02	0.123	0.75^Z^	0.76^Z^	0.69^Y^	0.02	0.020	0.75^YZ^	0.80^Z^	0.73^Y^	0.02	0.044
6 to 12 h	0.82^a,Z^	0.83^a,Z^	0.72^Y^	0.02	<0.001	0.80^a,Z^	0.82^a,Z^	0.73^Y^	0.02	<0.001	0.79^Z^	0.80^Z^	0.66^Y^	0.02	<0.001	0.79^Z^	0.84^Z^	0.71^Y^	0.02	<0.001
SEM	0.01	0.02	0.02			0.01	0.01	0.01			0.02	0.03	0.02			0.02	0.02	0.02		
p-value	0.01	0.01	0.34			0.02	0.02	0.22			0.29	0.11	0.38			0.61	0.10	0.67		
0 to 12 h	0.78^Z^	0.79^Z^	0.70^Y^	0.02	<0.001	0.77^YZ^	0.80^Z^	0.74^Y^	0.01	0.010	0.77^Z^	0.77^Z^	0.68^Y^	0.01	<0.001	0.77^YZ^	0.82^Z^	0.72^Y^	0.02	<0.001

Each least square mean represents 8 observations for each treatment. Data from collection for 4, 6, or 12 h were calculated based on collections in the six 2-h periods.

SEM, standard error of the mean.

1) WLP, waxy corn starch low-protein diet; CLP, corn starch low-protein diet; PLP, pea starch low-protein diet.

a,b Means in the same column with different superscripts differ among collection times (p≤0.05).

Y,Z Means in the same row with different superscripts differ among treatments (p≤0.05).
